# Failure to Eliminate Persistent *Anaplasma marginale* Infection from Cattle Using Labeled Doses of Chlortetracycline and Oxytetracycline Antimicrobials

**DOI:** 10.3390/vetsci8110283

**Published:** 2021-11-20

**Authors:** Andrew K. Curtis, Michael D. Kleinhenz, Tippawan Anantatat, Miriam S. Martin, Geraldine C. Magnin, Johann F. Coetzee, Kathryn E. Reif

**Affiliations:** 1 Department of Anatomy and Physiology, Kansas State University College of Veterinary Medicine, Manhattan, KS 66506, USA; akcrz4@vet.k-state.edu (A.K.C.); miriammartin@vet.k-state.edu (M.S.M.); gmagnin@vet.k-state.edu (G.C.M.); jcoetzee@vet.k-state.edu (J.F.C.); 2 Department of Clinical Sciences, Kansas State University College of Veterinary Medicine, Manhattan, KS 66506, USA; mkleinhe@vet.k-state.edu; 3 Department of Diagnostic Medicine/Pathobiology, Kansas State University College of Veterinary Medicine, Manhattan, KS 66506, USA; tippawan@vet.k-state.edu

**Keywords:** anaplasmosis, antibiotic, bacteremia, beef, Holstein, management, persistent infection, subclinical, tetracycline

## Abstract

Bovine anaplasmosis, caused by the intracellular rickettsial pathogen *Anaplasma marginale*, is the most prevalent tick-transmitted disease of cattle worldwide. In the U.S., tetracycline antimicrobials are commonly used to treat and control anaplasmosis. Oxytetracycline, administered by injection, is indicated for treatment of clinical anaplasmosis in beef and dairy cattle and calves. Chlortetracycline, administered orally, is indicated for control of active anaplasmosis infection in beef and nonlactating dairy cattle. Tetracyclines have been demonstrated to be effective for treating active anaplasmosis, but their ability to eliminate *A. marginale* at currently approved therapeutic doses or dosing regimens remains unclear. In the absence of approved dosing regimens for *A. marginale* clearance, a study was conducted to determine the effect of approved oxytetracycline and chlortetracycline indications on *A. marginale* bacteremia. Fifteen animals with persistent anaplasmosis were enrolled and divided into three treatment groups. Group 1 (*n* = 6) received oral chlortetracycline (1.1 mg/kg bodyweight) administered via hand-fed medicated feed for 60 consecutive days. Group 2 (*n* = 6) received injectable oxytetracycline administered subcutaneously at 19.8 mg/kg bodyweight three times in 3-week intervals. Group 3 (*n* = 3) served as an untreated control. After 60 days, bacteremia failed to permanently decrease in response to treatment. This result indicates that clearance of *A. marginale* is unlikely to be reliably achieved using currently approved tetracycline-based regimens to manage anaplasmosis.

## 1. Introduction

Bovine anaplasmosis is caused by the intracellular rickettsial hemobacteria *Anaplasma marginale* (*Am*) and is the most prevalent tick-transmitted disease of cattle worldwide [1]. Clinical signs include anemia, fever, weight loss, abortion, and death [2]. Disease can be divided into acute and persistent phases. During the acute phase of bovine anaplasmosis, bacteremia peaks, and the number of infected erythrocytes may be as high at 10^9^ per mL of whole blood [3]. Clinical signs of acute disease have been shown to appear once at least ~15% of an animal’s erythrocytes have been parasitized [4]. When compared to the acute phase, bacteremia is markedly lower during persistent bovine anaplasmosis. It has been reported that cyclic bacteremia can range from <10^4^ to 10^7^ parasitized erythrocytes per mL whole blood during persistent infection [5]. Anaplasmosis represents a significant obstacle for profitable beef production in the United States (U.S.), and losses associated with death, abortion, treatment, and control cost hundreds of millions of dollars per annum [6,7]. Seroprevalence is variable and depends on geographic region [7]. Among U.S. beef cattle, seroprevalence has been shown to range from 4.44% in Georgia [8] to 28.99% in Mississippi [9]. Importantly, low levels of bacteremia are maintained in surviving cattle [10]. Persistent, cyclical, *Am* bacteremia contributes to concomitant immunity among *Am* “carrier” cattle and overall disease endemic stability. Once infected, cattle often remain *Am* carriers for the duration of their life; thus, chronic anaplasmosis is the most common disease state among infected cattle [11]. Carrier animals represent a challenge to disease control as they can serve as asymptomatic reservoirs for vectored transmission of *Am* to naïve cattle [12]. The importance of anaplasmosis to the U.S. cattle industry is supported by its status as a national priority under the USDA ARS 2022–2027 National Program 103 Animal Health Action Plan in Component 3: Endemic Bacterial Diseases [13].

In the absence of a fully USDA-approved vaccine, anaplasmosis control has been predicated on the administration of tetracycline antimicrobials. The antimicrobial action of tetracyclines is generally bacteriostatic and is associated with reversible binding to the 30 S ribosomal subunit in susceptible bacteria [14]. Tetracyclines are widely used in both human and veterinary medicine, and various studies have suggested that antimicrobial resistance has emerged partially as a result of selective pressure exerted by expansive use in animals [15]. From 2009 to 2016, tetracyclines accounted for 70% by weight of all medically important antibiotics sold or distributed in the U.S. for use in food-producing animals [16]. Oxytetracycline (OTC) and chlortetracycline (CTC) are indicated for treatment [17] and control [18] of anaplasmosis, respectively. Currently, there is no antimicrobial approved for elimination or prevention of *Am*.

Injectable OTC is an U.S. Food and Drug Administration (FDA)-approved treatment, by or on the order of a licensed veterinarian, for anaplasmosis caused by *Am* [17]. Injectable OTC can be formulated in a sterile solution that contains 200 mg of OTC per mL of product. In this form, it can be delivered intramuscularly or intravenously at a dose of 1.1 mg/kg bodyweight (BW) per day for treatment of anaplasmosis. Where retreatment of anaplasmosis is impractical, a single injection of OTC can be intramuscularly or subcutaneously provided at 19.8 mg/kg BW. Presently, no OTC product available in the U.S. has a specific anaplasmosis label indication. Therefore, use of these products to treat anaplasmosis, though common, is off-label.

Oral CTC is approved by the FDA for controlling active anaplasmosis caused by susceptible strains of *Am* infecting beef and nonlactating dairy cattle [18]. CTC-medicated feed products can be hand-fed or provided free-choice within a drug-specific approved free-choice medicated feed formulation. The hand-fed FDA-approved dose of 1.1 mg CTC per kg BW is used to control active infection caused by *Am* susceptible to CTC. This regimen is indicated for beef cattle over 318 kg and requires a 48 h withdrawal period before animals are slaughtered. In addition, CTC can be fed to beef and nonlactating dairy cattle in free-choice feeds such as feed blocks or salt–mineral mixes as an aid in the control of active infection of anaplasmosis caused by *Am* susceptible to CTC. These free-choice feeds and mixes are formulated to provide a range of 1.1 to 4.4 mg CTC/kg BW. When formulated this way, CTC has no required withdrawal period. As of 1 January 2017, use of a CTC-medicated feed product (provided hand-fed or free-choice) requires a veterinarian–client–patient relationship and a veterinary feed directive from a licensed veterinarian.

Currently, no OTC injectable or CTC-medicated feed products are approved or labeled for elimination of persistent *Am* infections. Identification of a robust and reliable antimicrobial-based *Am* elimination regimen is highly sought by producers and veterinarians seeking to not only reduce the severity and duration of active anaplasmosis but also to resolve persistent infection among carrier animals. Elimination of persistent infection may not be appropriate for all animals. However, a reliable *Am* clearance protocol would be useful to cattle producers and veterinarians that want to clear infection from valuable stock for production or export purposes that require anaplasmosis-free cattle (e.g., embryo transfer cows, breeding stock), or other producers that are willing to maintain stringent biosecurity measures to prevent anaplasmosis introduction into their herd. Previous experiments have suggested that *Am* carrier clearance with tetracycline drugs ranges from successful [12,19] to unsuccessful [20]. Swift and Thomas [12] and Roby et al. [19] reported that OTC can eliminate the carrier state of anaplasmosis, but currently no OTC product has a specific label indication or FDA approval for this use. Similarly, previous studies have demonstrated that oral CTC antimicrobials are effective in controlling acute infection, but not for clearance of bacteria at approved doses [21,22]. The challenge of clearance may be further complicated by *Am* strain diversity. At least 43 strains of *Am* are known to circulate in the U.S. [23], and treatment efficacy is likely strain dependent [24]. In addition to potential variable susceptibility among *Am* strains, differences in drug dosing regimens (approved or experimental) can make direct comparisons of results among previous studies challenging [25,26]. Finally, standardized methods of determining clearance are not present across the published literature (e.g., PCR, serum agglutination and complement fixation assays, xenodiagnoses in splenectomized steers). Thus, rigorous studies are needed to evaluate and confirm the efficacy of antimicrobial treatment protocols, ideally using already approved drugs and drug dosages, to reliably clear *Am* infection caused by diverse *Am* strains.

Towards understanding the potential for tetracycline antimicrobials to eliminate *Am* infection, we conducted a study to evaluate *Am* bacterial level changes in persistently infected carrier cattle administered currently available OTC and CTC products. Specifically, the objective was to evaluate the ability of repeated oral CTC and injectable OTC administration to continually lower *Am* bacteremia to the point of clearance. Persistently infected steers were treated with oral CTC, delivered in feed each day for 60 consecutive days, or injectable OTC, delivered subcutaneously three times, once every 3 weeks, and their *Am* bacterial levels or status (infected versus uninfected) were compared to untreated controls and each other. While both tetracycline products share a similar mechanism of action, OTC was hypothesized to have a greater likelihood to eliminate *Am* infection due to greater drug dosages and dose timing, the latter expected to interrupt the normal cyclical bacteremia by reducing the opportunity for the emergence of new antigenic variants. Data gathered from this study will help inform dosing regimens and responsible antimicrobial stewardship when elimination of *Am* infection is desired by producers.

## 2. Materials and Methods

This study was conducted under approved Institutional Animal Care and Use Committee protocol #3959 on file in the University Research Compliance Office at Kansas State University, Manhattan, Kansas.

### 2.1. Animals

A cohort of 15 Holstein steers, aged approximately 30 months and weighing 807.9 +/− 57.1 kg (mean +/− SD), were enrolled in the study. All steers were confirmed persistently infected with *Am* by PCR prior to study enrollment. Steers had been experimentally infected with a field isolate of *Am* (Msp1a genotype M-F-F, sourced from a naturally infected *Am* carrier cow in Oklahoma in 2018) approximately 120 days prior to onset of this clearance study [27]. The M-F-F strain is a naturally circulating field isolate that has not been previously evaluated for antimicrobial sensitivity. As a requirement for enrollment, cattle had to be beyond the established withdrawal periods of any previously administered antimicrobial. Although not expected, all animals were monitored daily for signs of clinical anaplasmosis such as: anorexia (>24 h), pale mucus membranes, depression (>24 h), and/or increased respiratory rate (>60 breaths per min). Steers were co-housed in isolated dry lot pens away from study-unrelated cattle, fed a standard, balanced ration, and provided water ad libitum. To reduce arthropod vector transmission risk, steers were regularly treated with a permethrin-containing pour-on product (Ultra Boss^®^, Merck Animal Health, Kenilworth, NJ, USA) per label instructions. At study conclusion, animals were humanely slaughtered after all drug withdrawal periods had been satisfied.

### 2.2. Experimental Design

Steers were blocked by weight and randomly allocated into 1 of 3 treatment groups using the RAND function in a spreadsheet program (Microsoft Excel, Richmond, WA, USA). Study start was day 0, the first day of treatment administration. Group 1 steers (*n* = 6) were co-housed in the same pen and hand-fed CTC-medicated feed (Mid Kansas Cooperative Association, Moundridge, KS; CTC product: Pennchlor 50, Pharmgate Animal Health, Wilmington, NC, USA) mixed to provide 1.1 mg CTC/kg BW daily for 60 consecutive days. Feed containing CTC was manufactured once and was maintained in an outdoor bulk feed bin for the study duration. Group 2 steers (*n* = 6) were administered OTC (300 mg/mL, Noromycin 300 LA, Norbrook, Newry, UK) subcutaneously at 19.8 mg/kg once every 3 weeks for 6 weeks (at study days 0, 21, and 42). Finally, Group 3 steers (*n* = 3) received no antimicrobial treatment. Steers in Groups 2 and 3 were co-housed in the same pen and received an unmedicated version of the same feed ration as Group 1. On a weekly basis (±1 day if inclement weather), beginning 1 week prior to treatment and continuing for 10 weeks, blood samples were collected to evaluate bacteremia (*Am*/mL blood), OTC plasma concentration (parts per billion, ppb), and CTC plasma concentration (ppb). To collect blood samples and administer OTC to Group 2 steers, steers were led into and safely restrained in a cattle chute. Venipuncture utilized jugular or coccygeal veins. At each blood sampling time point, approximately 20 mL of blood was collected into a combination of evacuated tubes containing EDTA (for evaluation of *Am* bacteremia) or lithium heparin (for evaluation of OTC or CTC plasma concentration). Depending on the availability of personnel and handling equipment, Group 1 steers were normally sampled after CTC feeding. Steers were maintained until all drug withdrawal times were met.

### 2.3. Quantification of A. marginale Bacteremia

To determine *Am* bacteremia (*Am*/mL blood), a quantitative PCR assay (qPCR) targeting a portion of the single-copy *Am* Msp5 gene was used [28]. First, genomic DNA was extracted from 100 µL whole blood using the Quick DNA Miniprep Kit (Zymo Research, Irvine, CA, USA) according to manufacturer instructions. The resulting genomic DNA was eluted in 35 µL of DNA elution buffer. The PCR mixture was set up in 20 µL reaction volumes and included: 0.2 µM of each primer (*Am* msp5 F: ATA CCT GCC TTT CCC ATT GAT GAG GTA CAT, and *Am* msp5 R: AGG CGA AGA AGC AGA CAT AAA GAG CGT), 10 µL of SsoAdvanced Universal SYBR Green Supermix (Bio-Rad, Hercules, CA, USA), nuclease-free water, and 2 µL gDNA. Reaction cycling was performed using a CFX Connect Real-Time PCR System (Bio-Rad) with the following cycling parameters: 1 cycle of 98 °C for 2 min, followed by 40 cycles at 98 °C for 5 s, 60 °C for 5 s, and 74 °C for 15 s, and a final melt curve cycle of 65–95 °C with increasing 0.5 °C temperature steps at 10 s/step. Real-time qPCR data were visualized and analyzed using CFX Maestro Software v1.1 (Bio-Rad).

### 2.4. CTC and OTC Quantification

For the analysis of CTC, OTC was used as an internal standard. Similarly, for the analysis of OTC, CTC was used as an internal standard. CTC hydrochloride and OTC hydrochloride and phosphoric acid were sourced from Fisher Scientific (Thermo Fisher, Hampton, NH, USA) and stored at 4 °C until use. All LC-MS-grade solvents and phosphoric acid (85%) were sourced from Fisher Scientific. Ultrapure water (18 Ω) was obtained from an in-house Millipore UV-R system. Cleanup was performed using an HLB Prime µElution plate, 3 mg sorbent per well, from Waters Co. (Milford, MA, USA).

On the day of analysis, standard working solutions were prepared fresh from a stock solution of OTC at 100 µg/mL in methanol (free base). The following concentrations were prepared in aqueous phosphoric acid 4%: 1, 2.5, 5, 10, 25, 50, 100, 250 ppb. A solution of CTC (internal standard) at 50 ppb in aqueous phosphoric acid 4% was also prepared. Conversely, standard working solutions were prepared fresh from a stock solution of CTC at 100 ppb. The concentrations used for CTC were the same as those for OTC. A solution of OTC (internal standard) at 50 ppb in aqueous phosphoric acid 4% was prepared as well. Quality controls (QCs) for analysis of OTC were prepared in untreated bovine serum at the following OTC concentrations: 4.75, 47.5, and 95 ppb. For the analysis of CTC, QCs were prepared in untreated bovine serum at the following CTC concentrations: 7, 70, and 210 ppb.

Calibration standards, controls, samples, and QCs were prepared in a 48-well mixing plate. Calibration standards were prepared by mixing 100 µL of untreated serum with 100 µL of each standard. Negative controls were prepared by adding 100 µL of untreated serum to 200 µL of aqueous phosphoric acid 4%. Samples and QCs (100 µL) were mixed with 100 µL of aqueous phosphoric acid 4%. To each solution (except negative control), 100 µL of internal standard at 50 ppb was added. The plate was covered and shaken gently at 300 rpm on a platform for 10 min. The content of each well (300 µL) was loaded on the SPE µElution plate using a nitrogen processor to push the fluid through the sorbent. After washing with 300 µL of water–methanol (95:5), the CTC was eluted with 50 µL of acetonitrile–methanol (90:10) in a collection plate. To each well, 50 µL of aqueous 0.2% formic acid was added. The collection plate was covered with a cap-mat and shaken gently with a vortex mixer before analysis.

An ultra-high pressure liquid chromatography system (ULPC), Acquity H system, combined with a XEVO TQ-S triple mass spectrometer (Waters Co.) was used for analysis. The chromatographic separation was performed using the UPLC column Waters Acquity HSS T3 50 x 2.1 mm, 1.8 µm. The mobile phase consisted in a gradient of water with 0.1% formic acid (A) and acetonitrile (B) as follows: 0 min: 98% A; 1.5 min: 0% A; 2.0 min: 2.01 min: 98% A; 2.5 min: 98% A. The total run time was 2.5 min. The flow rate was set at 0.5 mL/min, the column temperature at 55 °C, and the autosampler compartment at 8 °C. The injection volume was 5 µL.

The acquisition was conducted by electrospray ionization in positive mode. The operating parameters for the mass spectrometer were as follows: the capillary voltage was 3.0 kV, source and desolvation temperatures were 150 °C and 600 °C, respectively, and the cone energy was set to 25 V. Nitrogen was used as the desolvation and cone gas at a flow rate of 1000 L/h and 150 L/h, respectively. Helium was used as the collision gas at a flow rate of 0.15 mL/min. Data acquisition and analysis were conducted using Waters MassLynx (Waters Co.) and TargetLynx (Waters Co.) software, respectively. The detection of OTC and CTC was performed using multiple reaction monitoring.

The lower limit of quantitation (LLOQ) was determined according to the FDA guidelines for the bioanalytical Method Validation Guidance for Industry [29] with a signal over noise ratio of > 5, precision of ≤ 20%, and accuracy between 40 and 120%. The LLOQ for CTC and OTC was determined to be 2.5 ppb (2.5 parts per billion, ppb) to 250 ng/mL (250 ppb). Linear regression was used with a weighing factor of 1/x. The calibration curve was linear from 2.5 ppb and accepted if the correlation coefficient was at least 0.99. The intra-day and inter-day precisions were <15%, and the accuracies for both CTC and OTC ranged from 80 to 100%.

### 2.5. Statistical Analyses

Statistical analyses evaluated the relationships between CTC and OTC concentrations (ppb) with bacteremia (*Am*/mL blood) over time. Bacteremia was log transformed prior to analysis. The outcome variables of bacteremia and CTC or OTC concentration were analyzed using a repeated measures test in SigmaPlot (SPSS Statistics, Chicago, IL, USA). Linear regressions were performed using JMP (SAS Institute, Cary, NC, USA) to examine relationships between drug concentrations and bacteremia. For all outcomes, statistical significance was set a priori at *p* < 0.05.

## 3. Results

### 3.1. Effect of CTC Treatment on A. marginale Bacteremia

**Figure 1:**
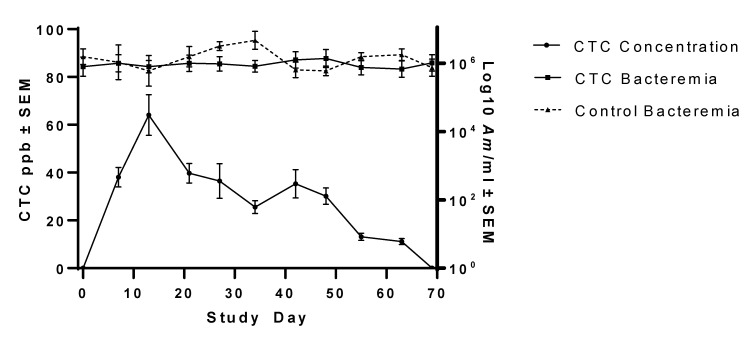
Changes in *Am* bacteremia in steers treated with chlortetracycline (CTC) for 60 days. CTC concentration, in parts per billion (ppb) ± standard error of mean (SEM), is plotted over time with bacteremia (*Am*/mL blood) ±SEM for animals treated daily with 1.1 mg/kg bodyweight CTC for 60 days. Untreated control steer mean *Am* bacteremia is included for comparison. CTC treatment was not found to have a significant (*p* > 0.05) effect on reducing *Am* bacteremia.

The ability of oral CTC to reduce *Am* bacteremia to the point of clearance was evaluated in Group 1 persistently infected steers. Summary statistics of *Am* bacteremia changes in CTC-treated steers and untreated steers are presented in Table 1. Group 1 animals treated daily with oral CTC, at 1.1 mg/kg BW, did not experience significantly decreased bacterial loads over the treatment period compared to mean starting *Am* bacteremia (*p* = 0.9980) (Figure 1). In addition, mean *Am* bacteremia (1.77 × 10^6^ copies/mL ± 2.64 × 10^5^ copies/mL) among Group 1 steers did not significantly differ from mean *Am* bacteremia (2.31 × 10^6^ copies/mL ± 4.78 × 10^5^ copies/mL) among Group 3 steers (untreated control) (*p* = 0.1834) during the study. Likewise, untreated control animals in Group 3 maintained persistent bacteremia that did not significantly differ over the study period (*p* = 0.3920).

Oral CTC treatment resulted in plasma CTC concentrations ranging between <2.5 and 84.6 ppb, with an average of 29.3 ± 2.6 ppb. Drug concentrations are summarized in Table 2. Plasma CTC concentrations peaked (mean 64.1 ± 21.0 ppb) 13 days after beginning treatment (Figure 1) before steadily declining for the remainder of the study. Linear regression indicated a poor (R^2^ = 0.0348, *p* = 0.1064) relationship between CTC concentration and bacteremia (Supplementary Figure S1).

### 3.2. Effect of OTC Treatment on A. marginale Bacteremia

**Figure 2:**
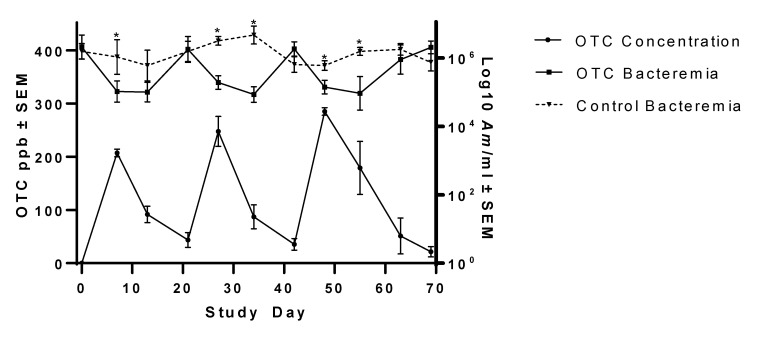
Changes in *Am* bacteremia in steers treated with multiple doses of oxytetracycline (OTC). OTC concentration, in parts per billion (ppb), is plotted over time with bacteremia (*Am*/mL blood) for animals administered 3 doses of OTC (19.8 mg/kg bodyweight) in 3-week intervals. Untreated control steer mean *Am* bacteremia is included for comparison. Asterisks (*) denote statistically significant differences in bacteremia between the OTC and untreated groups at the same time point (*p* < 0.05).

The ability of injectable OTC to reduce *Am* bacteremia to the point of clearance was evaluated in Group 2 persistently infected steers. Summary statistics of *Am* bacteremia changes in OTC-treated steers are presented in Table 1. Injectable OTC administered once every 3 weeks at study days 0, 21, and 42 at 19.8 mg/kg BW elicited a significant but transient reduction in *Am* bacteremia (Figure 2). OTC suppression of *Am* bacterial load was evident by each subsequent post-OTC treatment evaluation time point (~1 week later) and continued to decrease through at least another week, after which infection rebounded to near pre-treatment levels. The mean infection nadir observed post-OTC treatment was 5.25 × 10^5^ copies/mL ± 3.10 × 10^5^ copies/mL. Compared to Group 1 (CTC), Group 2 steers exhibited significantly lower *Am* bacteremia at study days 13 (*p* = 0.0168), 34 (*p* = 0.0103), 48 (*p* = 0.0094), and 55 (*p* = 0.0172). Compared to Group 3 (untreated control) steers, Group 2 steers exhibited significantly lower *Am* bacteremia at study days 7 (*p* = 0.0296), 27 (*p* = 0.0088), 34 (*p* = 0.0002), 48 (*p* = 0.0011), and 55 (*p* = 0.0087). Compared to baseline, Group 2 steers exhibited significantly lowered *Am* bacteremia at study days 7 (*p* = 0.0007), 13 (*p* = 0.0006), 27 (*p* = 0.0064), 34 (*p* = 0.0003), 48 (*p* = 0.0022), and 55 (*p* = 0.0005). However, these drops were transient, and by the third week, after each OTC treatment, mean bacteremia in Group 2 had returned to or exceeded baseline bacteremia or time-matched bacteremia levels in the untreated steers (e.g., at study day 69, Group 2 mean bacteremia was greater than the mean bacteremia of untreated steers).

Treatment resulted in plasma OTC concentrations between 9.3 and 420 ppb, with an average of 124 ± 13.4 ppb over the study period (days 7 to 70; day 7 is the first OTC plasma concentration evaluation time point post-initial treatment, and day 70 is 29 days post-final OTC treatment). Drug concentration data are summarized in Table 2. Plasma OTC concentrations peaked the week following each treatment (Figure 2), before declining. Peak plasma OTC concentrations averaged 247 ± 12.17 ppb. The relationship between drug concentrations and log-transformed bacteremia over time is illustrated in Supplementary Figure S2. A linear relationship (R^2^ = 0.2033, *p* = 0.0001) between OTC concentration and bacteremia was noted, suggesting that as the OTC concentration increases, bacteremia tends to decrease. This would suggest some susceptibility of the M-F-F *Am* strain to tetracyclines.

## 4. Discussion

This study investigated the ability of FDA-approved, commercially available tetracycline products to reduce *Am* bacteremia to the point of infection clearance in persistently infected steers. Groups of steers were either provided CTC daily at 1.1 mg/kg BW for 60 days, injected with OTC at 19.8 mg/kg BW three times at 3-week intervals, or received no treatment. Outcome measures included bacteremia, OTC concentration, and CTC concentration over time. Compared to pre-treatment *Am* bacteremia levels and untreated controls, the OTC treatment regimen significantly but transiently lowered *Am* bacteremia, but the CTC treatment regimen had no significant effect on *Am* bacteremia. By the end of the study, bacteremia levels had rebounded to near pre-treatment levels in both treatment groups and were similar to untreated control steer bacteremia levels. Currently, no antimicrobial drugs or products are approved for elimination of *Am* infection; use of the tetracycline products investigated in this study for *Am* clearance was for experimental purposes only.

In this study, Noromycin 300 LA, a commercially available injectable OTC product, failed to achieve *Am* bacterial clearance in steers with persistent anaplasmosis. It should be noted there is no specific FDA approval for the Noromycin 300 LA OTC formulation to be used in the context of bovine anaplasmosis. However, Noromycin 300 LA does include a label indication for use against disease caused by a wide range of susceptible Gram-negative bacteria. Further, the dose of 19.8 mg/kg BW is approved by the FDA for less concentrated OTC products (e.g., 200 mg/mL) where retreatment with injectable OTC is impractical [17]. Given the average weight of Group 2 steers (793 kg), use of Noromycin 300 LA required an average treatment volume of 52 mL instead of an average treatment volume of 79 mL had a 200 mg/mL OTC product been used. Further, the reduced volume required for Noromycin 300 LA reduced the total number of injections needed per treatment (six versus eight when using 10 mL/injection site as per manufacturer product administration directions). As this study was conducted in an experimental setting and limiting the number of injections was preferable in the interest of animal welfare, Noromycin 300 LA was used. Administration of injectable OTC resulted in reduced bacteremia (~26-fold reduction), with the greatest reduction observed 7–14 days post-treatment administration. Despite leading to a reduction in bacterial load, likely, in part, facilitated by the drug as well as the animal’s own immune response, *Am* infection was not cleared, rebounding to pre-treatment levels 7–14 days post-treatment bacteremia nadirs.

The OTC results in the present study are contradictory to previous work in which clearance was reportedly achieved using OTC dosing regimens ranging from 11 to 22 mg/kg BW given at intervals ranging from daily to weekly for between 5 and 14 days [19,30,31]. Other studies have achieved clearance through OTC injections at 20 mg/kg BW following 3–4 administrations at 3-day intervals [12,32]. As there is no standard protocol for determining bacterial clearance, it is possible that differences in methodology among previous experiments or the infecting *Am* strain contributed to different outcomes in this study versus previous studies. For example, Magonigle et al. [31] and Roby et al. [19] confirmed carrier clearance by subinoculating splenectomized blood harvested from OTC-treated *Am* carrier cattle at least 83 days after carrier cattle were treated. Özlem et al. [32] confirmed carrier clearance by harvesting blood from OTC-treated *Am* carrier cattle and observing a lack of organisms in stained blood smears. Although subinoculation of blood into a splenectomized calf is a robust way to investigate clearance, the available methodologies at that time to monitor infection (e.g., blood smears) had low sensitivity. Conversely, the present study relied on qPCR to quantify infection (direct visualization of *Am*-infected red blood cells on a thin blood smear is rare, and they not reliably detected during persistent *Am* infection). While the potential exists for molecular detection methods (e.g., qPCR) to detect genetic material from non-viable *Am* organisms, our results suggest that *Am* was not cleared. This is supported by the eventual rebound in the *Am* target sequence (also known as viable *Am*) over time in the OTC-treated animals and no significant reduction in the *Am* target sequence in the CTC-treated animals. If identification of the *Am* qPCR target sequence had fallen below the limit of qPCR detection, confirmation of infection elimination through xenodiagnosis (e.g., subinoculation of blood from the treated animal into a splenectomized naïve animal) could be used to confirm infection clearance. In a previous study where *Am* infection elimination was successful, the presence of the molecular assay target began to immediately wane and continued to decrease until falling below the limit of assay detection, after which infection elimination was confirmed by xenodiagnosis in a splenectomized calf [25]. It is also notable that previous studies investigating possible *Am* clearance protocols often used different *Am* strains, some of which may be more or less relevant when extrapolating which tetracycline-based *Am* elimination protocols may work best for contemporary *Am* strains. For example, previous work tested stains originating in Florida [33] and Oklahoma [20], and another [32] did not specify. Our results agree with a more recent study that reported clearance failure in naturally infected cattle using two doses of long-acting injectable OTC at 20 mg/kg [34]. Likewise, Coetzee et al. [20] reported clearance failure after injecting persistently infected steers with either one dose of OTC at 30 mg/kg, two doses of OTC at 30 mg/kg 5 days apart, or five doses at 22 mg/kg daily for 5 days. Data from the present study support that injectable OTC may be appropriate for reducing *Am* bacteremia to limit disease severity during acute anaplasmosis while the animal mounts an effective immune response but should not be considered reliable to achieve total *Am* clearance.

In the present study, peak serum OTC values were much lower than those measured in some previous trials. For example, Luthman and Jacobsson [35] found that injectable OTC peaked at between 1500 and 4000 OTC ppb in serum approximately 4 h after injection. One possible explanation for this discrepancy is the blood sampling schedule of the present study. In this case, blood was drawn at intervals much longer than the reported OTC half-life of 8 h [35]. Similarly, Xia et al. [36] reported peak plasma values of between 4000 and 10,000 ppb 6–9 h after injection. Cattle in the present study were sampled 7, 14, and 21 days after each OTC administration, and the observed drug concentrations likely reflected that regimen. Sampling animals with closer temporal proximity to treatment would have likely revealed higher peak OTC concentrations.

In the present study, oral CTC failed to clear *Am* or reduce the *Am* bacterial load in subclinical, persistently infected steers. The CTC dose of 1.1 mg/kg BW used in this study is approved by the FDA for control of active anaplasmosis. One potential reason for this result is that the FDA-approved dosing regimen (1.1 mg/kg BW per day) is not high enough to result in clearance of *Am* infection. Previous work has demonstrated *Am* clearance with CTC feeding when cattle were fed between 4.4 and 22 mg/kg BW daily [25]. Higher daily dosing in that study yielded higher mean CTC concentrations in plasma (85.3–518.9 ppb) than those measured in the present study (mean 29.3 ppb). Reinbold et al. [25] also gathered blood samples more frequently (sometimes as often as every 4 h) than the present study, likely contributing to differences in plasma CTC concentrations. However, at no time during the present study did more than 16.2 h, the oral CTC elimination half-life established for cattle [37], elapse between CTC feeding and blood sampling. Higher drug concentrations, achieved by CTC administration at levels higher than approved, may have contributed to greater bacteriostasis and subsequent *Am* clearance. In addition, the *Am* strain used in the present study differs from the Virginia isolate used by Reinbold et al. [25]. As with the OTC results, it is possible that genetic differences between isolates contributed to differences in susceptibility and overall results between studies.

A decline in plasma CTC concentration was noted in Group 1 steers during the course of their treatment regimen, suggesting that there may be drug stability issues in the medicated feed. The CTC-medicated feed used in the present study was manufactured in a single batch (received 3 days prior to study start) which was used for the duration of the study. Similar to the unmedicated feed, the CTC-medicated feed was stored in a waterproof outdoor bin during the study, as is the case on many commercial cattle operations. During the study, temperatures were cold to moderate, ranging from −2.2 to 25.6 °C, with 38.7 cm of precipitation [38]. It is conceivable that the diminishing steer plasma CTC concentration was due to loss of drug integrity over time, non-uniform initial feed ingredient mixing (less likely), or a non-uniform drug concentration due to settling of feed in the bin (Figure 1). Because the feed was not tested during the study, these possibilities are not able to be investigated.

Again, no CTC-medicated product is currently approved for prevention or elimination of *Am* infection in cattle. Oral CTC is approved for the control of active anaplasmosis. If disease control is interpreted as prevention of disease spread, oral CTC did not reduce *Am* bacteremia levels below untreated controls and therefore would be unlikely to reduce the risk of disease spread (e.g., via arthropod vectors or iatrogenic transmission) based on the assumption that treated animals would have lower bacteremia levels. Because *Am* can replicate in vector-competent tick species, ticks can effectively acquire *Am* from cattle with high or low levels of bacteria to subsequently transmit to naïve cattle [7]. If “control active anaplasmosis” is interpreted as prevention of clinical anaplasmosis, then it could be considered that CTC performed accordingly as no CTC-treated animal displayed any clinical signs of anaplasmosis; however, none of the untreated controls did either. The results presented here suggest that CTC, at the current approved dosages, would be unlikely to eliminate *Am* infection or even reduce the likelihood of transmission as *Am* bacteremia did not significantly vary from pre-treatment baseline or untreated controls during the 60 days of continuous treatment.

Despite the value it would have to the U.S. cattle industry, especially cow-calf and seed stock producers, a broadly effective, antimicrobial-based protocol to clear *Am* from carrier animals remains elusive. Presently, no OTC or CTC product or dosage has a label or approved indication for *Am* infection elimination from cattle. Data detailed here suggest that cattle producers and veterinarians should not anticipate or rely on labeled doses of OTC or CTC to eliminate *Am* in persistently infected cattle (nor are these products indicated for this purpose). Future efforts to identify a reliable *Am* infection elimination protocol could explore using current tetracycline products at different dosing frequencies or concentrations, or explore the utility of other drug products as tetracyclines are no longer the only antimicrobials approved for the treatment of bovine anaplasmosis in the U.S. As of 2020, the fluoroquinolone enrofloxacin has received conditional approval for the treatment of clinical anaplasmosis. Fluoroquinolone antimicrobials are generally bactericidal and exert their action through inhibition of topoisomerases [14]. As with CTC and OTC, enrofloxacin is not labeled for total *Am* infection clearance but has been shown to be effective at limiting mortality and anemia during acute anaplasmosis [39]. More research is needed to develop a robust and reliable antimicrobial-based protocol to eliminate persistent *Am* infection.

## 5. Conclusions

Long-term, persistent infection by *Am* remains a challenging aspect of bovine anaplasmosis management around the world. Treatment regimens designed to eliminate infection during this phase of disease are needed, but previous attempts have yielded varying results. Data from the present study indicate that U.S. FDA-approved dosages of either CTC or OTC are unlikely to eliminate *Am* infection. Although specific regulations on use may differ, the results from this study are broadly informative to other countries that rely on tetracyclines to combat bovine anaplasmosis. Future work is needed to evaluate the ability of antimicrobials to eliminate *Am* bacteremia and resolve the carrier state to promote economic potential and manage disease spread.

## Figures and Tables

**Table 1:** Summary of steer *Am* bacteremia values by study day and treatment group.

Day	Bacteremia (Log10 Copy/mL)																	
	CTC						OTC						Untreated Control					
	N	Mean	Median	Min	Max	SD	N	Mean	Median	Min	Max	SD	N	Mean	Median	Min	Max	SD
0	6	5.90	5.87	5.05	6.83	0.68	6	6.32	6.26	5.27	7.42	0.86	3	6.19	6.04	5.90	6.64	0.39
7	6	5.96	5.97	5.20	6.67	0.62	6	5.02	5.06	4.08	6.02	0.75	3	6.03	5.69	5.37	7.02	0.88
13	6	5.90	6.12	5.14	6.27	0.44	6	5.00	4.92	4.27	6.17	0.70	3	5.78	6.19	4.87	6.27	0.79
21	6	5.96	5.88	5.16	6.86	0.59	6	6.25	6.45	4.53	7.12	0.93	3	6.19	6.28	5.64	6.66	0.52
27	6	5.98	5.96	5.37	6.73	0.51	6	5.28	5.42	4.46	5.75	0.50	3	6.50	6.38	6.36	6.76	0.23
34	6	5.91	6.01	5.41	6.42	0.41	6	4.93	5.24	4.08	5.39	0.57	3	6.67	6.76	6.18	7.08	0.46
42	6	6.10	6.12	5.17	6.77	0.59	6	6.26	6.41	5.42	6.81	0.51	3	5.81	5.80	5.43	6.21	0.39
48	6	6.14	6.10	5.45	7.04	0.63	6	5.15	5.14	4.39	5.76	0.50	3	5.78	5.91	5.50	5.94	0.25
55	6	5.88	5.92	5.07	6.61	0.54	6	4.97	4.72	3.75	6.75	1.19	3	6.19	6.25	5.96	6.37	0.21
63	6	5.83	5.92	4.79	6.36	0.58	6	5.95	5.63	5.07	7.85	1.05	3	6.25	6.14	6.02	6.59	0.30
69	6	6.01	5.97	5.30	6.82	0.60	6	6.31	6.31	5.67	6.80	0.46	3	5.87	6.00	5.38	6.22	0.44

‘CTC’—chlortetracycline, ‘OTC’—oxytetracycline, ‘N’ number of animals, ‘SD’ standard deviation.

**Table 2:** Summary of steer plasma drug concentration levels by study day and treatment group.

Day	Drug Concentration (Parts per Billion—ppb)											
	CTC						OTC					
	N	Mean	Median	Min	Max	SD	N	Mean	Median	Min	Max	SD
0	6	0	0	0	0	0	6	0.28	0	0	1.70	0.69
7	6	38.00	38.80	25.40	52.30	10.14	6	207.10	205.95	185.50	228.90	17.18
13	6	64.05	67.90	32.20	84.60	20.95	6	91.58	82.50	58.90	160.10	38.28
21	6	39.67	35.05	31.80	56.60	10.09	6	43.77	33.30	14.20	103.10	34.39
27	6	36.43	29.30	22.40	70.10	17.92	6	247.50	247.55	141.40	328.50	69.26
34	6	25.58	24.75	16.20	35.70	6.66	6	87.12	65.50	39.60	179.10	55.43
42	6	35.28	30.75	17.80	53.90	14.48	6	35.37	27.05	11.60	87.60	27.23
48	6	30.15	28.25	21.10	42.90	8.39	6	285.30	284.35	263.30	314.60	17.36
55	6	13.10	12.10	10.30	19.80	3.65	6	179.00	143.65	89.40	420.00	122.83
63	6	11.10	11.50	7.20	16.00	3.17	6	51.18	48.10	32.80	77.20	16.71
69	6	0	0	0	0	0	6	21.45	21.35	9.30	39.10	10.91

## Data Availability

All data generated or analyzed during this study are included in the published article or Supplementary Files.

## Supplementary Materials

The following are available online at https://www.mdpi.com/article/10.3390/vetsci8110283/s1, Figure S1: Regression of bacteremia and CTC concentration, Figure S2: Regression of bacteremia and OTC concentration.
