# Supplementary material for: Failure to Eliminate Persistent Anaplasma marginale Infection from Cattle Using Labeled Doses of Chlortetracycline and Oxytetracycline Antimicrobials
Source: Vet Sci. 2021 Nov 20;8(11):283. doi: 10.3390/vetsci8110283 (PMC8621018; doi:10.3390/vetsci8110283)
Supplement: Supplementary file 1 [file vetsci-08-00283-s001.zip › vetsci-1429335-supplementary.pdf]

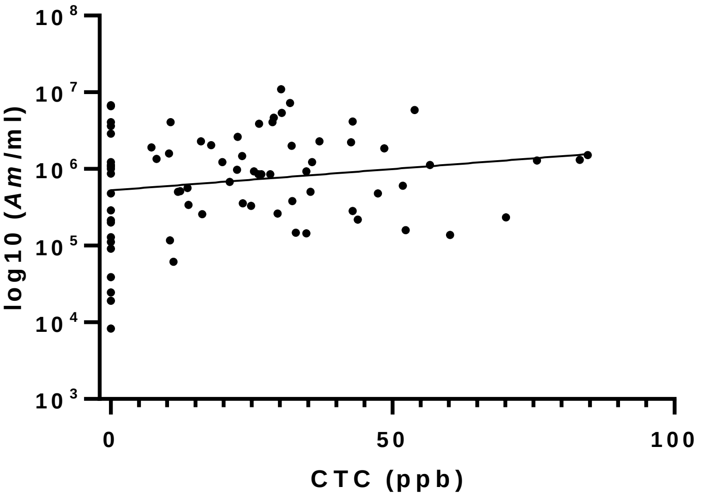

Supplementary Figure S1. **Relationship between *Am* bacteremia and drug concentration in steers treated with chlortetracycline (CTC) for 60 days**. Bacteremia (*Am*/mL blood) for animals treated with CTC is plotted by CTC concentration, in parts per billion (ppb). A straight line denotes linear regression (R^2 = 0.0348, P = 0.1064).

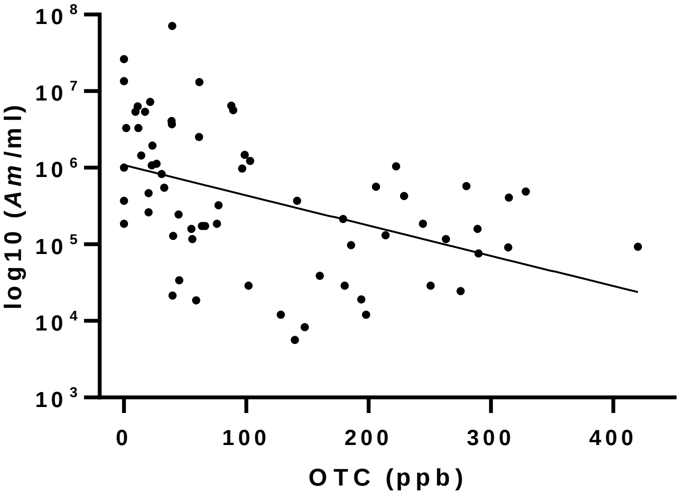

Supplementary Figure S2. **Relationship between *Am* bacteremia and drug concentration in steers treated with oxytetracycline (OTC)**. Bacteremia (*Am*/mL blood) for animals treated with OTC is plotted by OTC concentration, in parts per billion (ppb). A straight line denotes linear regression (R^2 = 0.2033, P = 0.0001).
